# Structure of Rat Ultrasonic Vocalizations and Its Relevance to Behavior

**DOI:** 10.1371/journal.pone.0014115

**Published:** 2010-11-29

**Authors:** Nobuaki Takahashi, Makio Kashino, Naoyuki Hironaka

**Affiliations:** 1 NTT Communication Science Laboratories, Nippon Telegraph and Telephone Corporation, Atsugi-shi, Japan; 2 Core Research for Evolutional Science and Technology, Japan Science and Technology Agency, Kawaguchi-shi, Japan; 3 Graduate School of Humanities, Kwansei Gakuin University, Nishinomiya-shi, Japan; Texas A&M University, United States of America

## Abstract

Rats are known to emit ultrasonic vocalizations (USVs). These USVs have been hypothesized to hold biological meaning, and the relationship between USVs and behavior has been extensively studied. However, most of these studies looked at specific conditions, such as fear-inducing situations and sexual encounters. In the present experiment, the USVs of pairs of rats in ordinary housing conditions were recorded and their features were examined. Three clusters of USVs in the 25-, 40-, and 60-kHz range were detected, which roughly corresponded to fighting, feeding, and moving, respectively. We analyzed sequential combinations of two or more clusters using a state transition model. The results revealed a more specific correspondence between the USVs and behaviors, suggesting that rat USV may work as a type of communication tool.

## Introduction

Rats emit ultrasonic vocalizations (USVs) in response to various situations [1]. For example, long (>0.3 s) 22-kHz USVs are emitted in aversive contexts, such as after defeat in an aggressive encounter [2], [3], presentation of a cue predicting foot shock [4], and in sexual contexts, such as after ejaculation [5]. In contrast, short (<0.3 s) 50-kHz USVs are emitted during play [6], copulation [7], and aggression [2].

USVs are thought to induce or modulate behaviors: 40-kHz USVs by isolated infant rats induce maternal and retrieval behavior in adult female rats [8], [9]; 50-kHz USVs emitted by male rats induce solicitation behavior in female rats [10], [11]; and 22-kHz USVs reduce aggressive behavior of dominant male rats [2].

Moreover, USVs are thought to be indices of affective states [1]. Appetitive stimuli facilitate 50-kHz USVs (social stimuli [6], [12], food cues [13]) and aversive stimuli facilitate 22-kHz USVs (presence of a predator [14], handling by unfamiliar humans [15], footshock cues [4], [13], and food cue extinction [13]). Electrophysiological or pharmacological treatments induce either 50- or 22-kHz USVs, depending on whether their nature is appetitive or aversive [13], [16]–[22].

These findings suggest that USVs in rats work as a communication tool that carries emotional and/or environmental information. If so, a pair of rats might emit USVs even in an ordinary housing situation. Since conventional studies have recorded USVs in specific experimental settings, such as encounters with an intruder or an estrous female, the question of whether rats emit USVs in non-controlled situation has not been answered.

Moreover, if USVs in rats carry information, not only a single call but also a sequence of calls could provide some biological meaning. Indeed, recent studies have suggested such a possibility. A study using mouse USVs, analyzed the features of a “syllable”, a unit of sound separated by silence from another sound unit, and temporal sequencing of “syllables” [23] and demonstrated that the specific sequence of “syllables” can be regarded as a “song” in the sexual context. In studies using rats, a sequence of calls is known as a step [24]–[25], which is an instantaneous change to a higher or lower frequency. Rapid frequency oscillations are known as trills. These two calls are frequency-modulated (FM) calls. Wright et al., [25] classified 50-kHz USVs on the basis of spectrograms. These previous studies dealt with sequences of calls as combinations. In order to analyze USVs more fully, a useful approach would be to deal with the components of USV combinations as units of USVs.

In this study, we attempted to categorize the USVs of pairs of rats in ordinary housing conditions and examined the features of USV “syllables” using a state transition model and analyzed relationships between USV “syllables” and behaviors. From this examination, we found that sequences of USV “syllables” correspond to specific behaviors (feeding, moving, and fighting). We automatically identified three clusters of USV calls: cluster 1 calls corresponded to 22-kHz calls; cluster 2 calls corresponded to the flat and lower components of the step; and cluster 3 calls corresponded to the trill, upward ramp, and higher components of the step. Moreover USV calls evoked at feeding situation were virtually cluster 2 calls only. A part of cluster 2 calls have a function similar to that of “food calls” found in rhesus monkeys.

Our findings suggest that rat USVs and their “syllables” have biological meaning.

## Results

### Behaviors and clusters of USVs

After recording, we investigated audio recording data 50 ms at a time, and when USVs were emitted, we analyzed video recording data, which were synchronized with the audio recordings. The video recordings showed that all USVs were emitted during locomotor activity; that is, USVs were not emitted when neither rat was engaged in locomotor activity (i.e., sleeping).

**Figure 1:**
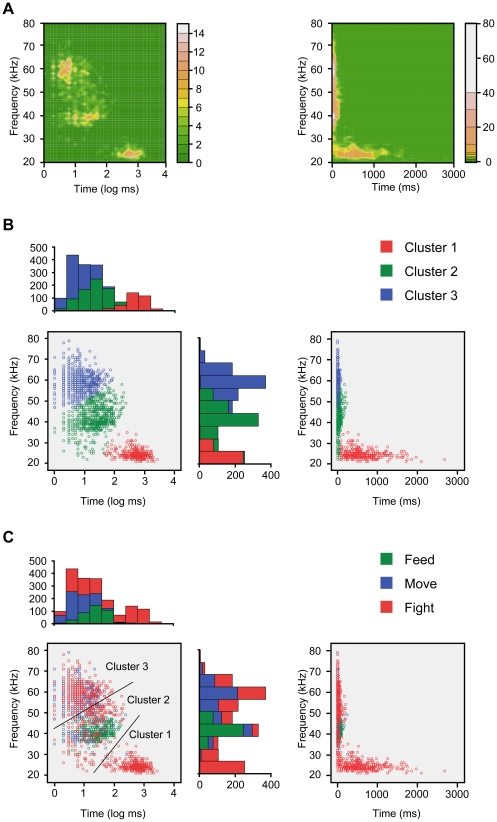
Scattar plots of cluster and behavior. (A) Power graph and (B) scatter plot of frequency vs. duration of USV calls. A total of 1785 USV calls from the three pairs were aggregated. USV calls were separated into three cluster groups. (C) The same data shown in (B) was color-coded by behavioral categories. Left: log transformed data. Right: nonlog transformed data.

We identified three clusters of USV calls using a two-step cluster analysis. These clusters differed in frequency and in duration (Figure 1B). Cluster 1 was characterized by low frequency (24.56±2.18 kHz) and long duration (628.70±414.45 ms; log_10_: 2.69±0.33), cluster 2 by moderate frequency (41.78±5.88 kHz) and moderate duration (31.18±32.40 ms; log_10_: 1.29±0.43), and cluster 3 by high frequency (59.18±4.91 kHz) and short duration (9.16±10.08 ms; log_10_: 0.80±0.36). In both frequency and duration, each cluster was significantly dissimilar from the average (Bonferroni adjustment applied, p<.05). Moreover, USV calls were categorized by reference to Wright et al. [25]. The residual analysis showed that cluster 3 comprised FM calls, such as “upward calls” and “trills”. In contrast, cluster 2 comprised constant-frequency calls (“flat”) (Table 1). Note that combination calls (e.g., steps) were decomposed to components (e.g. flat and short)

**Table 1:** Frequency of USVs by clusters and USVs categories.

	Cluster 1	Cluster 2	Cluster 3
Upward	0 (7.1)**	13 (15.5)	26 (16.3)**
Downward	1 (0.9)	1 (2)	3 (2.1)
Flat	10 (104.6)**	454 (228.2)**	109 (240.1)**
Short	0 (148.5)**	229 (323.8)**	584 (340.7)**
Trill	0 (5.3)*	5 (11.6)*	24 (12.2)**
Inverted U	0 (0.7)	2 (1.6)	2 (1.7)
22-kHz	315 (58.8)**	7 (128.3)**	0 (134.9)**

Values in parentheses are expected ones. Residual analyses of clusters and USVs categories showed a significant difference between observed and expected values (*p<.05, **p<.01).

**Figure 2:**
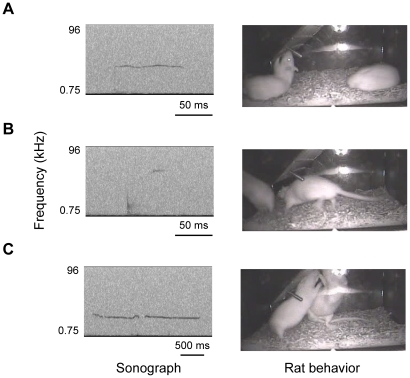
USV characteristics and rat behavior. Representative sonogram and image during (A) feeding (gnawing chow on the food hopper), (B) moving (galloping; right rat), and (C) fighting (mutual upright posture).

The video recordings revealed that there were three clear behavioral categories: feeding, mainly consisting of gnawing a piece of chow in the paws and gnawing at chow embedded in the cage lid (Figure 2A); moving, mainly consisting of walking, trotting, galloping, jumping, and rearing (Figure 2B); and fighting, mainly consisting of allogrooming, upright posture, aggressive posture, submissive-supine posture, and attack jumps (Figure 2C). However, our data do not distinguish between rat pairs individually; the concurrent activities of a pair were mainly fell into the same behavioral categories (i. e., feeding, moving, and fighting). When a few concurrent activities occurred across categories (5%), we determined which rat emitted USVs on the basis of evoked timing.

We found these ultrasonic calls were frequently separated by short (7.42±12.70) silent intervals. Though the traditional terminology defines a “syllable” as a unit of sound separated from other sound units by silence [26], in the following state transition diagrams, we incorporated these short silences as a part of a “syllable”.

### Relationship between behaviors and USVs

Figure 1C shows the data from Figure 1B color-coded by behavioral categories. As shown in the figure, feeding and moving were limited to a small area, but fighting covered a large area.

The chi-square test and residual analysis showed that rat USVs assigned to behavioral categories were not homogeneous (χ^2^ (4) = 1020.73, p<.01). That is, cluster 1 calls were emitted dominantly during fights, cluster 2 calls were emitted dominantly during feeding, and cluster 3 calls were emitted dominantly during movement (Table 2). However, there was a substantial overlap in clusters 2 and 3. There, calls were emitted both during feeding and movement. Thus, we proceeded to a sequential analysis in which two or more calls and short (<50 ms) intervals of silence were treated as “syllables” (Table 3).

**Table 2:** Frequency of USVs by clusters and behaviors.

	Feed	Move	Fight
Cluster 1	1 (70.1)**	6 (100.1)**	319 (155.8)**
Cluster 2	375 (153.0)**	136 (218.3)**	200 (339.8)**
Cluster 3	8 (160.9)**	406 (229.6)**	334 (357.4)*

Values in parentheses are expected ones. Residual analyses of clusters and behaviors showed a significant difference between observed and expected values (*p<.05, **p<.01).

**Table 3:** Matrix of transition probabilities of rat syllables.

	Cluster 1	Cluster 2	Cluster 3	Silence	End
Start	0.07	0.42	0.50	- -	- -
Cluster 1	0	0.22	0.02	0.44	0.33
Cluster 2	0.01	0.02	0.07	0.56	0.34
Cluster 3	0.00	0.03	0.02	0.58	0.37
Silence	0.05	0.29	0.65	- -	- -

Transition probabilities of 333 syllables composed of more than one USV call were calculated. These probabilities were calculated from frequencies of transition from one state to another or for repetition of the same state. Four cells are blank because these pairs did not occur.

### Characteristics of “syllables” in each behavioral category

#### Feeding

**Figure 3:**
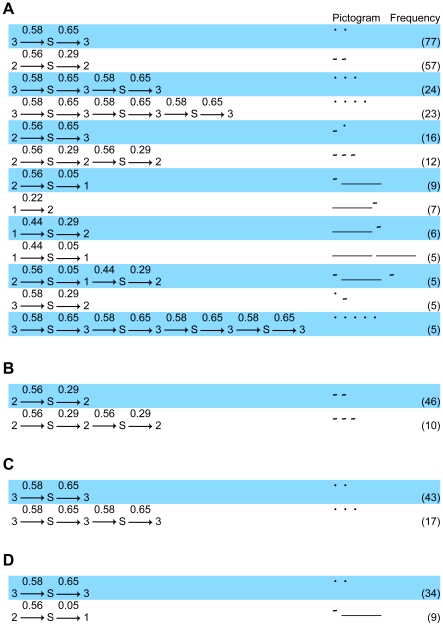
Frequently appearing rat syllables. (A) Ten syllables with the highest frequency from all data. The characters sandwiching the arrows indicate the cluster number or silence (denoted as “S”). The numbers above the arrows are probabilities for the transition. At the pictogram column, “_____”, “-”, “ ˙”, and “ ” represent clusters 1, 2, 3, and silence, respectively. (B, C, and D) Top 2 syllables with the highest frequency in feeding (B), moving (C), and fighting (D).

**Figure 4:**
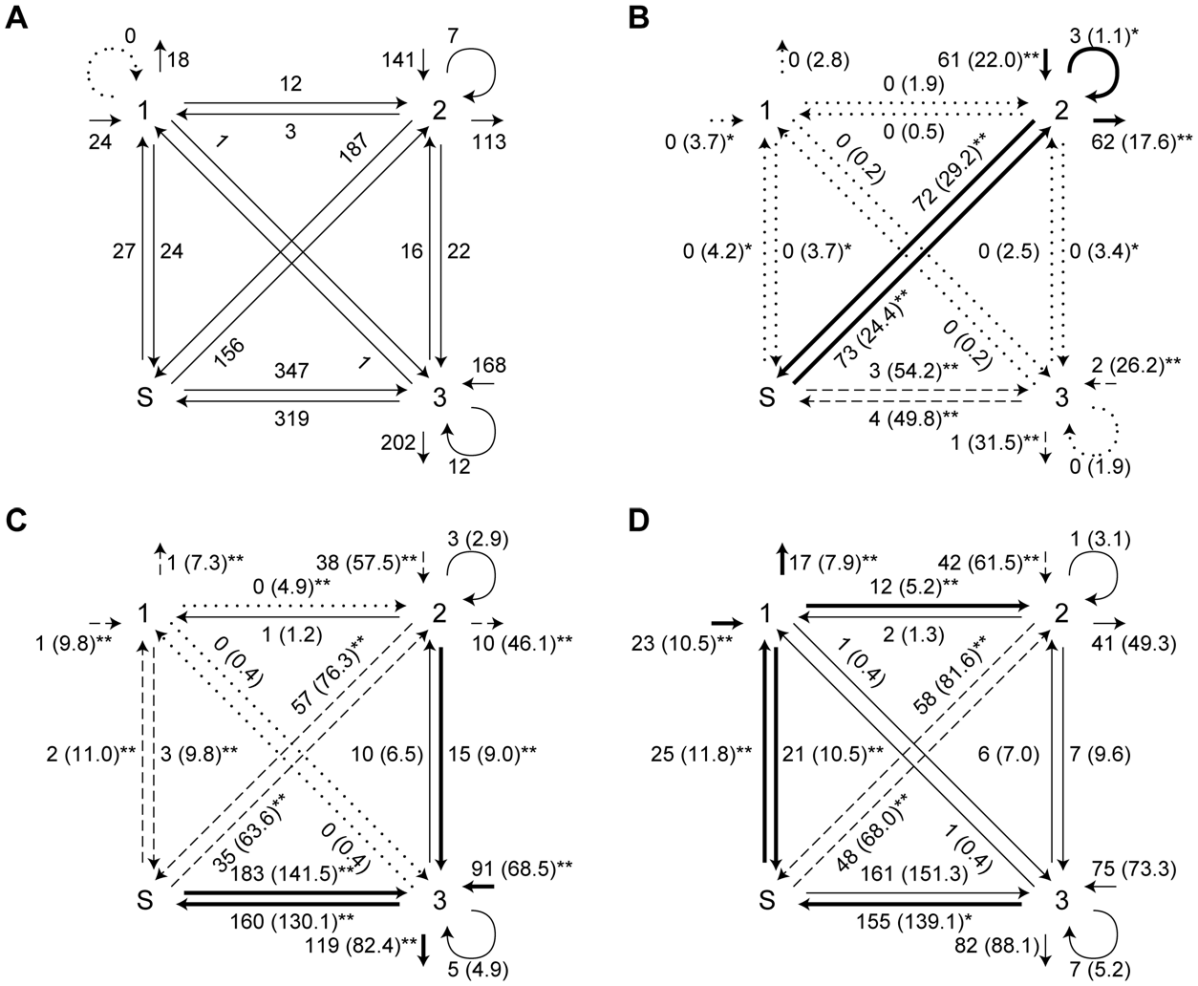
State transitions of USV calls and behavior. (A) Four-state transition model. The states correspond to three clusters (1, 2, and 3) and silence (S). The numbers bordering the arrows are frequencies for the transition. (B), (C), and (D) show frequencies for transitions in feeding, moving, and fighting. Values in parentheses indicate expected ones. Asterisks indicate results of residual analyses of clusters and behaviors (*p<.05, **p<.01), excuding repetition of cluster 1 without silence because this transition was not observed in all data. Bold arrows mean the observed value was significantly larger than expected value; dashed arrows mean the observed value was significantly smaller than expected; dotted arrows mean no transition was observed; and thin arrows mean not significant.

During feeding (Figure 3B and 4B), the syllables were characterized by starting with a cluster 2 call (p<.01), finishing with a cluster 2 call (p<.01), repetition of a cluster 2 call (p<.05), transition from a cluster 2 call to silence (p<.01), and transition from silence to a cluster 2 call (p<.01). Starting and ending with a cluster 3 or 1 and other transitions were significantly rare or absent.

#### Moving

During movement (Figure 3C and 4C), the syllables were characterized by starting with a cluster 3 call (p<.01), finishing with a cluster 3 call (p<.01), transition from a cluster 3 call to silence (p<.01), transition from silence to a cluster 3 call (p<.01), and transition from a cluster 2 call to a cluster 3 call (p<.01). The frequencies of repetition of cluster 2 or 3 as well as transition from a cluster 2 call to cluster 1 and from a cluster 3 call to cluster 2 were not significant. Starting and ending with cluster 1 or 2 and other transitions were significantly rare or absent.

#### Fighting

During fights (Figure 3D and 4D), the syllables were characterized by starting with a cluster 1 call (p<.01), finishing with a cluster 1 call (p<.01), transition from a cluster 1 call to silence (p<.01), transition from silence to a cluster 1 call (p<.01), transition from a cluster 3 call to silence (p<.05), and transition from a cluster 1 call to a cluster 2 call (p<.01). In this case, the frequency of starting with cluster 2 as well as transition from cluster 2 to silence and from silence to cluster 2 was significantly rare (p<.01). Other sequences were not significant.

## Discussion

We identified three clusters of USVs: about 25 kHz (from 20–30 kHz), 40 kHz (from 35–50 kHz), and 60 kHz (>50 kHz). Among them, a 25-kHz call (cluster 1) is thought to match the well-known so-called 22-kHz call. A characteristic of this call is a long duration (300–3,000 ms). The frequency of the 25-kHz call also matches the traditionally known 22-kHz call (18–32 kHz).

However, 40-kHz (cluster 2) and 60-kHz (cluster 3) calls need some explanation, because they did not accord categories identified in the conventional literature. A 50-kHz call (length, 20–80 ms; frequency, 35–70 kHz) and a 40-kHz call have been described [1]. However, a 40-kHz call is a “distress” call emitted by infant rats separated from their mothers. Moreover, a detailed sonographic analysis has shown that the actual averaged peak frequency of this “distress” call is higher than 40 kHz [27]. Since we did not use isolated pups in this study, this cluster 2 call is not considered to match the “distress” call.

Because our classification of USVs is different from conventional studies' with the aim of examining transition probabilities, cluster 2 calls corresponded to the flat and lower components of the step and cluster 3 calls corresponded to the trill, upward ramp, and higher components of the step. Moreover, a cluster 2 (40 kHz) call is often related to feeding, it could not be detected in an experimental situation without food consumption not reward. The absence of a “feeding” call might lead to reducing bimodal peaks and then to locating the dominant frequency at about 50 kHz.

Each of the three clusters of USV calls found is considered to correspond to a specific behavior: cluster 1 (25 kHz) to fighting, cluster 2 (40 kHz) to feeding, and cluster 3 (60 kHz) to moving. This suggests that the phonetic components of USVs themselves carry biological meaning with respect to different kinds of situations. Moreover, if either rat's behavior was offensive upright, cluster 2 and 3 were dominant, rather than cluster 1 (Table S1). Thus, in this study, USVs evoked in fight situations were a mixture attacker and defender calls.

However, the correspondence was not definite and substantial overlaps were found, except for cluster 1. A cluster 1 call approximately corresponded to fighting and a cluster 2 call corresponded to feeding. However, an almost-concurrent occurrence of cluster 1 and 2 calls did not mean that the rats engaged in both fighting and feeding at the same time. Actually, this combination was a transition from cluster 1 to cluster 2 in a short period and was emitted by a rat showing submissive-supine posture.

Thus, a sequence analysis of these clusters regarding them as “syllables” is important. In most cases, calls of two or more clusters were connected by short intervals of silence. Thus, we incorporated these silent intervals into the syllabic analysis.

Probabilities of transition were not homogeneous. For example, a transition from silence to cluster 3 was the most frequently observed. In contrast, repetition of cluster 1 never occurred (Figure 4A). This specificity suggests that sequential combinations of two or more cluster calls have biological significance, though we did not eliminate the possibility that such sequential combinations arose from articulatory restriction, as has been pointed out in rats and mice [23], [28].

The behavioral analysis underscored this notion. For example, for moving, transition from cluster 2 to cluster 3 that was identified as step up [25] was frequently observed. For fighting, transition from cluster 1 to cluster 2 was frequent. Repetition of a cluster 2 call with a short silent interval found in feeding behavior was unique and emitted rarely in other situations. These “syllables” might be similar to “food calls” found in rhesus monkeys [29], [30].

However, since this study was the first step of the “syllabic” analysis of rat USVs, there were several transitions whose behavioral meaning was unclear. For example, a transition of “cluster 3 - silence - cluster 3” was found in moving as well as in fighting. Further analysis of more intimate structures will reveal critical factors differentiating the biological meaning of these ambiguous sequences. Further study will be necessary to clarify the biological meaning of rat USVs. Playbacks of probabilistically deviant USVs may have efficacy for a functional of USVs. For example, if playbacks of feeding syllables influence rat feeding behavior and playbacks of probabilistically deviant syllables do not influence it, this would suggest that feeding syllables have functional meaning as an emotional contagion [31], [32].

In conclusion, paired rats emitted three kinds of USVs and their combined “syllabic” calls corresponded to feeding, moving, and fighting behaviors. These calls may work as a type of communication tool.

## Materials and Methods

### Ethical considerations

The experiment was conducted in accordance with the guidelines for animal experiments in research institutes issued by the Japanese Ministry of Education, Culture, Sports, Science and Technology and was approved by the ethical committee of the Japan Science and Technology Agency (permit numbers: 17-Department of Planning and Coordination, Office of Basic Research, Japan Science and Technology Agency-17).

### Subjects

Male Sprague-Dawley rats purchased from CLEA Japan, inc (Jcl: SD) at eight- weeks old were used. A male rat was paired with another male and housed throughout the acclimation and experiment period. Three pairs were used in the recording. Each pair was housed in a polycarbonate cage (26 cm width×43 cm depth×20 cm height) in an environmentally controlled rearing system (EBAC-L, CLEA Japan Inc.) where temperature and humidity were kept constant (23+/−1°C and 50+/−5%, respectively) and external sound sources were shut out. The inside of the system was illuminated from 8:00 AM to 8:00 PM. Externanl sources of light were shut out. All recordings were conducted in this system. Tap water and standard rat laboratory chow (CE-2, CLEA Japan, inc.) were available ad libitum.

### USV and behavior recording

Pairs were reared at least three days before recording. Recordings were conducted during the dark period. USVs were recorded with microphones (1/4″ Microphone Type 4938, Brüel & Kjær, Nærum, Denmark) and preamplifiers (1/4″ Microphone Preamplifier 2633, Brüel & Kjær, Nærum, Denmark). Behavior was simultaneously recorded with USVs by means of a night-vision camera (CAR-B3106, Keiyo Techno, Tokyo, Japan). Microphones were suspended close to the lid of the cage. The night-vision camera was placed 20 cm from the cage. Recordings from 9:00 to 11:00 PM were used for analysis, thus 6 hours data (3 pairs×2 hours) were used in this study.

### Analysis

Sounds were digitized at 192 kHz and stored to disk using Audacity software. The duration of the USV from the sonogram and the highest peak in the power spectrum were measured. A two-step cluster analysis (SPSS 13.0J) was performed with frequency and duration to reveal clusters among individual USVs. The number of clusters was automatically determined on the basis of Schwarz Bayesian Criterion, and as the type of distance measure, the log-likelihood criterion was used. In the two-step clustering algorithm, the first step is the formation of preclusters. In the second step, the standard hierarchical clustering algorithm on the preclusters is used (See [33] for details). Because the clustering algorithm is based on a distance measure needs a normal distribution of data, a common logarithmic transformation of duration time was used. Behaviors were observed on movie files on the basis of the time at which USVs were emitted.

## Supporting Information

Table S1 Frequency of USVs by clusters and detailed behaviors. Refer to Mitchell's criteria [34]; observed detailed behavioral indexes were as follows: locomotion, rearing, approach, follow (to the conspecific), nose and investigate, attempt mount, sniff genitalia, aggressive groom, aggressive posture, attack, bite, offensive sideways (broadside approach to conspecific), offensive upright (upright with head orientated towards the conspecific), pull (bite with moving backwards), defensive sideways (as offensive sideways but with the head oriented away from the conspecific), defensive upright (as offensive upright but with the head oriented away from the conspecific), submit, attend, crouch, flag and evade, retreat, under food hopper (escape to under food hopper), digging, drinking, eating, licking (own body fur), scratching, shaking, washing (wipe face), and stretching. A slash mark means that the two rats in a pair showed different behaviors. A plus sign means that one rat showed two behavioral indexes.
(0.13 MB DOC)
