# Supplementary material for: Structure of Rat Ultrasonic Vocalizations and Its Relevance to Behavior
Source: PLoS One. 2010 Nov 29;5(11):e14115. doi: 10.1371/journal.pone.0014115 (PMC2993931; doi:10.1371/journal.pone.0014115)
Supplement: Table S1 — Frequency of USVs by clusters and detailed behaviors. Refer to Mitchell's criteria [34]; observed detailed behavioral indexes were as follows: locomotion, rearing, approach, follow (to the conspecific), nose and investigate, attempt mount, sniff genitalia, aggressive groom, aggressive posture, attack, bite, offensive sideways (broadside approach to conspecific), offensive upright (upright with head orientated towards the conspecific), pull (bite with moving backwards), defensive sideways (as offensive sideways but with the head oriented away from the conspecific), defensive upright (as offensive upright but with the head oriented away from the conspecific), submit, attend, crouch, flag and evade, retreat, under food hopper (escape to under food hopper), digging, drinking, eating, licking (own body fur), scratching, shaking, washing (wipe face), and stretching. A slash mark means that the two rats in a pair showed different behaviors. A plus sign means that one rat showed two behavioral indexes. (0.13 MB DOC) [file pone.0014115.s001.doc]

Table S1. Frequency of USVs by Clusters and Detailed Behaviors

| Behavioral Indexes | Cluster 1 | Cluster 2 | Cluster3 |
| --- | --- | --- | --- |
| locomotion | 2 | 81 | 247 |
| locomotion / rearing | 0 | 6 | 12 |
| locomotion / submit | 0 | 1 | 10 |
| locomotion / retreat | 0 | 0 | 2 |
| locomotion / under food hopper | 0 | 1 | 0 |
| rearing | 2 | 15 | 64 |
| rearing / submit | 2 | 1 | 1 |
| rearing / attend | 0 | 0 | 4 |
| rearing / crouch | 0 | 0 | 2 |
| rearing / retreat | 0 | 0 | 2 |
| rearing / under food hopper | 4 | 1 | 1 |
| rearing / eating | 0 | 28 | 6 |
| rearing / follow | 1 | 1 | 0 |
| bite / under food hopper | 9 | 2 | 0 |
| offensive sideways / attack | 0 | 0 | 2 |
| offensive upright | 0 | 0 | 4 |
| offensive upright / offensive upright | 8 | 42 | 27 |
| offensive upright / defensive sideways | 0 | 0 | 3 |
| offensive upright / defensive upright | 5 | 5 | 6 |
| offensive upright / submit | 3 | 5 | 4 |
| offensive upright / retreat | 1 | 1 | 0 |
| offensive upright / licking | 0 | 1 | 1 |
| offensive upright / shaking | 1 | 0 | 0 |
| pull | 0 | 0 | 2 |
| deffensive sideways | 2 | 0 | 0 |
| deffensive sideways / attend | 0 | 0 | 2 |
| deffensive upright | 7 | 1 | 0 |
| submit | 4 | 6 | 2 |
| submit / retreat | 5 | 1 | 1 |
| crouch | 0 | 2 | 0 |
| crouch / submit | 2 | 3 | 1 |
| flag and evade | 0 | 0 | 1 |
| flag and evade / submit | 1 | 0 | 0 |
| approach | 2 | 6 | 66 |
| approach / rearing | 1 | 0 | 1 |
| approach / offensive upright | 0 | 0 | 5 |
| approach / defensive sideways | 2 | 1 | 0 |
| approach / submit | 1 | 1 | 3 |
| approach / attend | 0 | 0 | 1 |
| approach / retreat | 0 | 0 | 3 |
| approach / under food hopper | 5 | 0 | 0 |
| approach / digging | 1 | 4 | 3 |
| approach / shaking | 0 | 0 | 1 |
| retreat | 1 | 17 | 46 |
| retreat / defensive sideways | 1 | 0 | 2 |
| retreat / defensive upright | 1 | 2 | 2 |
| under food hopper | 75 | 5 | 7 |
| under food hopper / retreat | 0 | 3 | 0 |
| under food hopper + digging | 2 | 0 | 1 |
| under food hopper + drinking | 3 | 1 | 0 |
| digging | 0 | 0 | 1 |
| drinking | 0 | 1 | 1 |
| drinking / submit | 4 | 1 | 0 |
| eating | 1 | 336 | 7 |
| eating / locomotion | 0 | 5 | 0 |
| eating / digging | 0 | 2 | 0 |
| eating / drinking | 0 | 1 | 0 |
| eating / licking | 0 | 3 | 0 |
| licking | 0 | 3 | 0 |
| scratching | 0 | 1 | 0 |
| scratching / submit | 2 | 0 | 0 |
| shaking | 0 | 6 | 7 |
| shaking / defensive upright | 0 | 0 | 3 |
| washing | 0 | 0 | 1 |
| stretching | 0 | 0 | 1 |
| follow | 0 | 0 | 3 |
| follow / retreat | 1 | 3 | 39 |
| follow / under food hopper | 0 | 2 | 4 |
| nose and investigate | 0 | 1 | 7 |
| nose and investigate / rearing | 0 | 2 | 0 |
| nose and investigate / defensive sideways | 0 | 0 | 2 |
| nose and investigate / defensive upright | 1 | 0 | 0 |
| nose and investigate / submit | 3 | 2 | 0 |
| nose and investigate / retreat | 0 | 0 | 1 |
| attempt mount | 0 | 0 | 3 |
| attempt mount / offensive upright | 0 | 0 | 4 |
| sniff genitalia | 0 | 1 | 2 |
| sniff genitalia / submit | 5 | 2 | 0 |
| aggressive groom / defensive upright | 24 | 0 | 0 |
| aggressive groom / submit | 56 | 39 | 42 |
| aggressive groom / under food hopper | 6 | 1 | 12 |
| aggressive posture / submit | 40 | 31 | 16 |
| aggressive posture / crouch | 2 | 2 | 3 |
| aggressive posture / retreat | 1 | 0 | 0 |
| aggressive posture / washing | 2 | 0 | 0 |
| attack | 4 | 5 | 14 |
| attack / rearing | 0 | 2 | 10 |
| attack / offensive upright | 4 | 3 | 0 |
| attack / defensive sideways | 6 | 0 | 0 |
| attack / submit | 6 | 6 | 8 |
| attack / crouch | 0 | 0 | 1 |
| attack / retreat | 0 | 2 | 3 |
| attack / under food hopper | 4 | 3 | 3 |
| attack / eating | 0 | 1 | 0 |
| attack / follow | 0 | 0 | 2 |

Refer to Mitchell’s criteria [34], observed detailed behavioral indexes were as follows: locomotion, rearing, approach, follow (to the conspecific), nose and investigate, attempt mount, sniff genitalia, aggressive groom, aggressive posture, attack, bite, offensive sideways (broadside approach to conspecific), offensive upright (upright with head orientated towards the conspecific), pull (bite with moving backwards), defensive sideways (as offensive sideways but with the head oriented away from the conspecific), defensive upright (as offensive upright but with the head oriented away from the conspecific), submit, attend, crouch, flag and evade, retreat, under food hopper (escape to under food hopper), digging, drinking, eating, licking (own body fur), scratching, shaking, washing (wipe face), and stretching. A slash mark means that the two rats in a pair showed different behaviors. A plus sign means that one rat showed two behavioral indexes.
